# Sophisticated Framework between Cell Cycle Arrest and Apoptosis Induction Based on p53 Dynamics

**DOI:** 10.1371/journal.pone.0004795

**Published:** 2009-03-10

**Authors:** Hiroyuki Hamada, Yoshihiko Tashima, Yu Kisaka, Kazunari Iwamoto, Taizo Hanai, Yukihiro Eguchi, Masahiro Okamoto

**Affiliations:** 1 Laboratory for Bioinformatics, Graduate School of Systems Life Sciences, Kyushu University, Fukuoka, Japan; 2 Bio-architecture Center, Kyushu University, Fukuoka, Japan; Center for Genomic Regulation, Spain

## Abstract

The tumor suppressor, p53, regulates several gene expressions that are related to the DNA repair protein, cell cycle arrest and apoptosis induction, which activates the implementation of both cell cycle arrest and induction of apoptosis. However, it is not clear how p53 specifically regulates the implementation of these functions. By applying several well-known kinetic mathematical models, we constructed a novel model that described the influence that DNA damage has on the implementation of both the G2/M phase cell cycle arrest and the intrinsic apoptosis induction via its activation of the p53 synthesis process. The model, which consisted of 32 dependent variables and 115 kinetic parameters, was used to examine interference by DNA damage in the implementation of both G2/M phase cell cycle arrest and intrinsic apoptosis induction. A low DNA damage promoted slightly the synthesis of p53, which showed a sigmoidal behavior with time. In contrast, in the case of a high DNA damage, the p53 showed an oscillation behavior with time. Regardless of the DNA damage level, there were delays in the G2/M progression. The intrinsic apoptosis was only induced in situations where grave DNA damage produced an oscillation of p53. In addition, to wreck the equilibrium between Bcl-2 and Bax the induction of apoptosis required an extreme activation of p53 produced by the oscillation dynamics, and was only implemented after the release of the G2/M phase arrest. When the p53 oscillation is observed, there is possibility that the cell implements the apoptosis induction. Moreover, in contrast to the cell cycle arrest system, the apoptosis induction system is responsible for safeguarding the system that suppresses malignant transformations. The results of these experiments will be useful in the future for elucidating of the dominant factors that determine the cell fate such as normal cell cycles, cell cycle arrest and apoptosis.

## Introduction

The tumor suppressor, p53, is a transcription factor that frequently exhibits an abnormal synthesis in malignant cells [1]. Ultraviolet (UV) irradiation and ionization radiation can fractionate the DNA double-stranded structure, and activate the p53 synthesis process that subsequently induces the DNA damage signal transduction system. The activated p53 regulates several gene expressions that are related to the DNA repair protein, cell cycle arrest and apoptosis induction [2]. The primary role of p53 in the cell cycle mechanism is to prevent cells from reaching the mitotic phase before the DNA damage is repaired. p53 activates gene expressions for p21, 14-3-3 sigma, growth arrest and DNA damage factor 45 (GADD45), among others, and thus, interferes in the cell cycle checkpoint mechanisms to arrest the cell cycle progression [3], [4]. In contrast, the primary role that p53 plays in the induction of apoptosis is to cause cells with severe DNA damage to initiate programmed cell death, which therefore acts to suppress the proliferation of malignant cells. p53 regulates gene expressions for Bcl-2, and Bcl-2 associated×protein (Bax), among others, and can cause initiation of a cascade reaction of the intrinsic apoptosis induction system [5]. Thus, by activating p53, normal cells have the ability to implement both cell cycle arrest and the induction of apoptosis. However, it is not clear how p53 specifically regulates the implementation of these functions.

**Figure 1:**
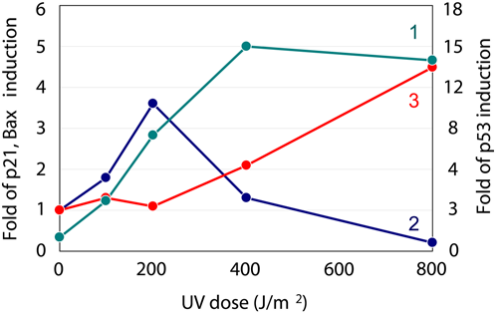
Relationship between the synthesis of biochemical species and the UV dose. The solid lines 1, 2 and 3 were p53, p21 and Bax, respectively. These profiles were extracted from the data presented in previously published report [6].

To verify the relationship between cell cycle arrest and the induction of apoptosis, Li *et al.* treated a Human Dermal Fibroblast clump with UV irradiation and observed that there were several protein levels that were associated with the DNA damage [6]. Figure 1 schematically shows their experimental results, which elucidate the relationship between the synthesis of biochemical species and the UV dose. A low UV dose (application of less than 200 J/m^2^) promoted synthesis of both p53 and p21, but was not linked to the synthesis of Bax. In contrast, a high UV dose (between 200 and 400J/m^2^) promoted the synthesis of both p53 and Bax, but decreased p21 as compared to that observed with the low UV dose. In the case of an excessive UV dose (more than 400J/m^2^), the synthesis of Bax, p53 and p21 exhibited more activation, slight inactivation and suppression, respectively, as compared to that seen for the high UV dose. Since p21 and Bax are activated with the low UV dose and the high UV dose, respectively, these findings imply that normal cells are able to implement either cell cycle arrest or the induction of apoptosis depending upon the level of DNA damage. The question that remains to be answered is whether or not cells can preferentially activate one or both of these functions. To further elucidate the p53 dynamics in detail, Lev Bar-Or *et al.* treated a clump of human breast cancer epithelial MCF-7 cells with excess gamma ray irradiation (5 Gy). They observed that there was a time course associated with several of the protein levels related to the DNA damage signal transduction system [7]. The MCF-7 cell aborts the implementation of apoptosis induction because of mutations in the genes for caspase3 [8]. However, these cells can be used to examine the protein level time courses based on the severe DNA damage [9]. Lev Bar-Or *et al.* reported that the time courses for the protein level for both p53 and Mdm2 showed an oscillation behavior after excess gamma ray irradiation. They also found that the oscillatory phase of Mdm2 was impeded as compared to that seen for p53. Moreover, based on these findings, Lev Bar-Or *et al.* constructed a kinetic mathematical model for the DNA damage signal transduction system, and demonstrated that the negative feedback effects that occur between p53 and Mdm2 were responsible for the oscillations generated. In addition, Lahav *et al.* used a similar experimental model to observe several oscillations for both p53 and Mdm2 on the MCF-7 single cell, and demonstrated that these oscillations were caused by an intrinsic mechanism [10]. These biological findings suggest there is possibility that the oscillation of p53 plays an important role in the implementation of either the cell cycle arrest or the induction of apoptosis. The validity of this postulation should be able to be inclusively confirmed by employing a numerical analysis that evaluates the impact of the p53 dynamics on both the cell cycle arrest and the apoptosis induction. Although the kinetic mathematical model for both of these systems has been previously reported by Aguda, Bagci and Stefan, respectively [11]–[13] (Supporting information Figures S1, S2, S3), as of yet there is no effective kinetic mathematical model that can be used to verify the above-mentioned speculation.

In the current study, we attempted to numerically elucidate the effect of p53's dynamic behavior in the implementation of both the cell cycle arrest and the apoptosis induction. To achieve this, we first constructed a novel kinetic mathematical model that described the influence that DNA damage has on the implementation of both the cell cycle arrest and the apoptosis induction via its activation of the p53 synthesis process. Next, in order to identify the critical conditions that lead to the implementation of the cell cycle arrest and/or the apoptosis induction, we used the proposed model to examine the relationships present during the p53 activation, the cell cycle arrest and the apoptosis induction. Finally, we examined the contributions of p53's dynamic behavior on the implementation of both the cell cycle arrest and the apoptosis induction. The results of these experiments might be useful in the future for developing novel therapeutic systems for tumor tissue and for elucidating of the dominant factors that determine the destiny of cells (cell fate) such their roles in normal cell cycles, cell cycle arrest and apoptosis.

## Results and Discussion

**Figure 2:**
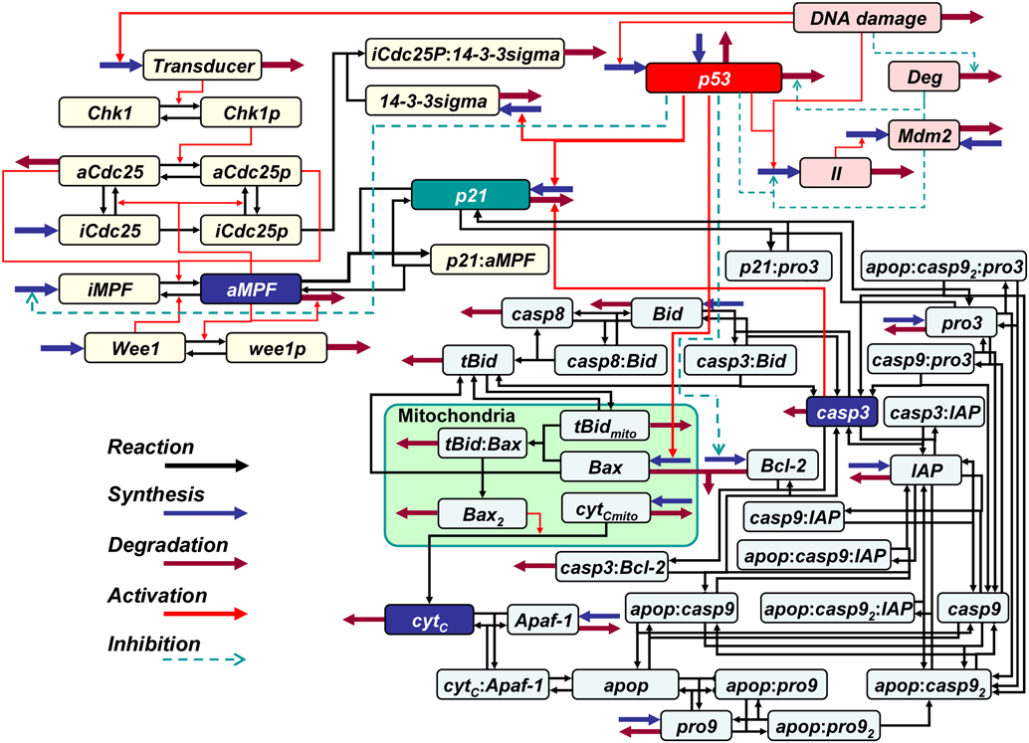
Proposed model reaction scheme. Blue and russet thick arrows represent the synthetic and degradation processes, respectively. Black and red arrows show the reaction and activation, respectively, while the dashed arrow indicates suppression (see main text and supporting information for detail). Dependent variables are shown in Supporting information Table S1.

**Figure 3:**
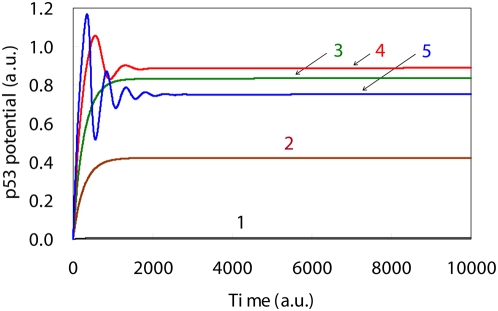
Simulation results for the p53 level time courses based upon DNA damage levels. Time course of the p53 levels in the presence of high DNA damage showed an oscillation behavior. The initial DNA damage levels, which are shown by the solid lines 1, 2, 3, 4 and 5, were: 0.0, 0.001, 0.002, 0.003 and 0.004, respectively.

**Figure 4:**
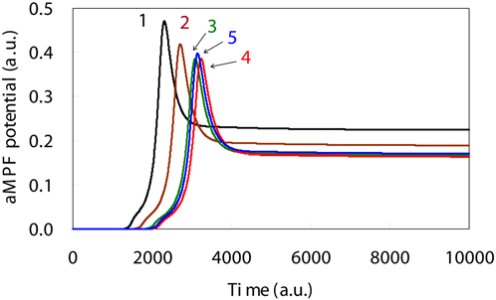
Simulation results for the aMPF level time courses based upon DNA damage levels. Time course of aMPF levels in the presence of high DNA damage showed an activation time lag as compared to that seen with low DNA damage. The initial DNA damage levels, which are shown by the solid lines 1, 2, 3, 4 and 5, were: 0.0, 0.001, 0.002, 0.003 and 0.004, respectively.

**Figure 5:**
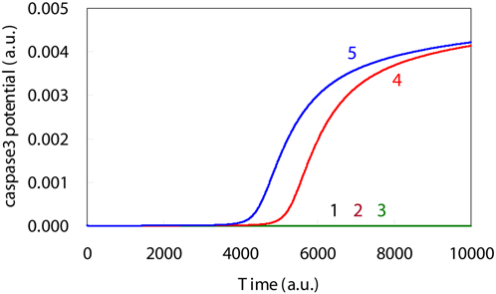
Simulation results for the caspase3 level time courses based upon DNA damage levels. Time course of the caspase3 levels in the presence of high DNA damage indicated there was an activation, which implied that there was a complete induction of apoptosis. The initial DNA damage levels, which are shown by the solid lines 1, 2, 3, 4 and 5, were: 0.0, 0.001, 0.002, 0.003 and 0.004, respectively.

**Figure 6:**
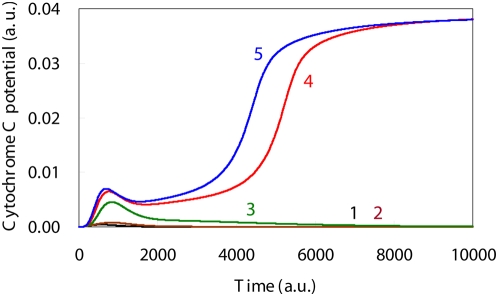
Simulation results for the Cytochrome C level time courses based upon DNA damage levels. Time course of Cytochrome C levels indicated there was a bifurcation that occurs when there is induction of apoptosis. The initial DNA damage levels, which are shown by the solid lines 1, 2, 3, 4 and 5, were: 0.0, 0.001, 0.002, 0.003 and 0.004, respectively.

We constructed a novel kinetic mathematical model (proposed model) for which the p53 oscillation system simultaneously interferes in the G2/M phase cell cycle and in the intrinsic apoptosis induction (Figure 2). The proposed model consisted of 32 dependent variables and 115 kinetic parameters. More detailed information related to each reaction process of the proposed model can be seen in the Supporting information tables and figures (Supporting information Tables S1, S2, S3, S4, Figures S4, S5, S6, S7, S8, S9, S10). As shown in Figure 2, the p53 activated by the DNA damage was responsible for the regulation of the synthesis of p21, 14-3-3 sigma, inactive MPF, Bcl-2 and Bax (Supporting information Table S2). The initial set of conditions for the biochemical species (Supporting information Table S3) was decided based on upon previously reported values [7], [11]–[12]. A simultaneous numerical optimization technique [14] that used a WinBEST-KIT [15] was performed in order to determine the set of kinetic parameters (Supporting information Table S4). Our results indicated there was little discrepancy from the data presented in previously published reports [12], [16]–[18]. Thus, we used the proposed model to simultaneously calculate the protein level time courses, and to verify the effects of the initial DNA damage level on the dynamic behaviors of both the cell cycle arrest and the apoptosis induction (Figures 3, 4, 5, 6).

When the initial level of DNA damage was equal to 0.0 (no DNA damage), the p53 level time course was constant and very low (Figure 3). In contrast, when the initial level was set to between 0.001 and 0.002 (low DNA damage), the p53 showed a sigmoidal behavior with time (Figure 3). And, when the initial level was set to between 0.003 and 0.004 (high DNA damage), the p53 showed an oscillation behavior with time (Figure 3). In addition, the p53 peak levels increased with increases in the initial DNA damage levels. Li *et al*. showed that when DNA damage increased, there was activation of the p53 synthesis process [6]. Lev Bar-Or *et al.* and Lahav *et al.* both reported that cells with serious DNA damage exhibited an oscillation behavior for all of the p53 time course levels [7], [10]. The p53 time courses are shown in Figure 3 and were in good agreement with the qualitative biological findings. Thus, these findings indicate that it is the various dynamic behaviors of p53 that make it possible for the diversity in the synthetic rates of key proteins such as p21, 14-3-3 sigma, Bcl-2 and Bax to occur.

Lev Bar-Or *et al*. treated a clump of human breast cancer epithelial MCF-7 cells with gamma ray irradiation, and observed several time courses of p53 [7]. When the level of gamma ray was equal to 0.3 Gy and 5.0 Gy, p53 showed a sigmoidal and oscillation behavior with time, respectively. The biological findings of these gamma ray irradiations are identified as follows: 0.3 Gy is no noticeable symptoms and 5.0 Gy is the lethal dose for 50% of exposed population (LD50) [19]. Lev Bar-Or moreover, constructed a mathematical model for the DNA damage signal transduction system (Supporting information Figure S4), and verified a relationship of p53 dynamic behavior to intensity of DNA damage [7]. When the initial DNA damage was set to 0.8, the time course of p53 showed a sigmoidal behavior corresponding to an above mentioned biological finding for 0.3 Gy. Meanwhile, when the initial DNA damage was set to 1.0, the time course of p53 showed an oscillation behavior corresponding to a biological finding for 5.0 Gy. In our model, low DNA damage and high DNA damage qualitatively produced a sigmoidal and oscillation behavior of p53 with time, respectively (Figure 3). Based on a comparison of our result with several biological findings reported by Lev Bar-Or *et al*. and Lahav *et al*. [7], [10], the numerical analysis for which initial level of DNA damage in our model was equal to 0.001 corresponded to the biological findings for 0.3 Gy, and that for initial level of DNA damage was 0.004 corresponded to that for 5.0 Gy. Moreover, Vilenchik reported that the irradiation induces double-strand breaks with a yield of 30 per cell per Gy [20]. Therefore, an increase of initial level of DNA damage in our model increases both the number of double-strand breaks and the lethal rate.

With an increase in the initial level of DNA damage, there was a delay in the peak time of the aMPF level, along with a decrease in the peak level of aMPF (Figure 4). The delay of aMPF implies there was arrest of the cell cycle. Bunz showed that cells with DNA damage are able to interrupt the progression of the cell cycle so that the DNA damage can be repaired [21]. Theron *et al.* reported that an increase in the level of the DNA damage extends the retardation of the cell cycle progression and decreases the peak levels of aMPF [22]. As seen in Figure 4, the relationship between the initial level of DNA damage to the dynamic behavior of aMPF was in good agreement with the qualitative biological findings. Therefore, we used a kinetic mathematical model that integrated Aguda's model with the p53 oscillation system in order to verify the relationship of p53's dynamic behavior with the cell cycle arrest.

Irradiation of cells using a high UV dose causes serious DNA damage as compared to that seen for a low UV dose. Based on previous findings, it was expected that increased UV dose levels would result in greater delays of the aMPF peak time. However, Li *et al.* reported that the cells exposed to low UV doses more effectively activated p21 synthesis as compared to that seen for the other UV dose levels [6]. Since p21 acts as a suppressor of the activation of aMPF, this biological finding suggests that the cell cycle continues in cells with serious DNA damage before the DNA damage can be sufficiently repaired. As shown in Figure 4, there was little difference noted between aMPF peak levels when the initial levels of DNA damage were set to 0.003 and 0.004. To evaluate the efficiency of p53 on aMPF activation, the area under the curves for p53 (AUC_p53_) were measured for each of the initial DNA damage levels (Table 1). The maximum AUC_p53_ was identified in a p53 oscillation for which the initial DNA damage level was set to 0.003. As shown in Figure 3, the amplitude was larger and the duration was shorter for the p53 oscillation when the initial DNA damage level was set to 0.004 versus 0.003. In this study, the AUC_ p53_ was maximal with an initial DNA damage level set to 0.003, and when set at this level, caused a maximal disturbance of the aMPF activation. This finding predicts that there is a mechanism that causes normal cells to become cancerous, i.e., there is possibility that the p53 oscillation behaviors of cells with grave DNA damage allow for the cell cycle progression without sufficiently repairing the DNA damage. In addition, the alterations of the aMPF peak times were in good agreement with the associated AUC_p53_ between the time ranges of 0 and 2000 (Table 1). According to the biological findings [22], simulated results between 0 and 2000 in Figure 4 can be regarded as the dynamic behaviors of the G2 phase. Therefore, the AUC_p53_ for the G2 phase can be used to determine the delay that will occur in the aMPF activation, which is the dominant factor for the implementation of the G2/M phase cell cycle arrest.

**Table 1:** Relationship between initial DNA damage and AUC_p53_.

Initial DNA damage level	aMPF peak time	AUC_p53_			
		Time period shown in Fig. 2 (time)			
		0–2,000	0–3,000	0–4,000	0–5,000
0.000	2310	12	19	26	33
0.001	2710	730	1152	1574	1997
0.002	3090	1445	2279	3113	3947
0.003	3235	**1694**	**2581**	**3468**	**4356**
0.004	3150	1501	2251	3001	3753

Bold formatting cells indicate the maximum AUC_p53_ for each of the time range integrations.

DNA damage occurred at time = 0.

When the initial levels were set to a low DNA damage level, the simulated results for the apoptosis induction system indicated that the synthesis of caspase3 was suppressed during the entire simulation period (Figure 5). In contrast, when there were high DNA damage levels, the caspase3 time course exhibited sigmoidal behavior that appeared after the aMPF activation seen in Figure 4, which implies there was an implementation of apoptosis (Figure 5). In addition, there was a significant discrepancy for the Cyt_C_ time course between the high DNA damage and the low DNA damage levels (Figure 6). As a result of the high DNA damage, the dynamic behavior of p53 caused sequential activation of the Bax, Cyt_C_ and Caspase3 synthesis process, which qualitatively supports the intrinsic apoptosis induction system mechanism [23].

Li *et al.* reported that as compared to low DNA damage levels, the high DNA damage promoted synthesis of both p53 and Bax while decreasing that of p21 [6]. Kohn and Pommier showed that p53 activates the synthesis of Bax while suppressing Bcl-2 synthesis, which is associated with the Bax degradation process [5]. Chowdhury also demonstrated that Bax promotes an excretion process that causes the transfer of Cyt_C_ from the mitochondria to the cellular cytoplasm [24]. These biological findings suggest that for the intrinsic apoptosis induction to occur, there needs to be a drastic increase in the synthesis rate of Bax in order to wreck the equilibrium between Bcl-2 and Bax. As seen in Figure 6 the Cyt_C_ time course exhibits a bifurcation that indicates there is an induction of apoptosis after the primary peak. We measured the AUC_p53_ during the time range when the integration was equal to 500, which corresponded to Cyt_C_'s primary peak time (Table 2). The results indicated that the AUC_p53_ increased with increases in the initial DNA damage level (Table 2), although there was little discrepancy with regard to the amount of Cyt_C_ activation (Figure 6). Moreover, since the p53 time course for the high DNA damage levels showed an oscillation behavior, the p53 peak level for high DNA damage was much greater than that seen for the low DNA damage (Figure 3). The tremendous activation of p53 with oscillation dynamics made it possible to dramatically increase the synthetic rate of Bax, which contributed to the induction of apoptosis. Therefore, the apoptosis induction occurs shortly after the activation of p53, and moreover, the p53 oscillation behaviors are an indication of the induction of the intrinsic apoptosis. Zhang reported that when the subtype of p53 related to the apoptosis induction shows an oscillation behavior, the apoptosis is subsequently induced [25]. Moreover, Geva-Zatorsky suggested that the oscillation plays a general role in stress or damage response [9]. These findings are in good agreement with the above-mentioned results. However, since Zhang and Geva-Zatorsky applied the simplified model to evaluate an effect of p53 dynamics in the apoptosis induction, it was difficult to elucidate the detailed mechanism for which the p53 dynamics lead the apoptosis induction and the cell cycle arrest. Thus, our proposed model is able to elucidate the sophisticated framework that occurs between the G2/M phase cell cycle arrest and the induction of apoptosis, and is useful for evaluating the effect of p53 dynamics that determine the cell fate as compared to other conventional models.

**Table 2:** Relationship between AUC_p53_ and p53 peak level.

Initial DNA damage level	AUC_p53_ 0–500 (time)	maximum p53 level
0.000	1	0.006
0.001	115	0.355
0.002	229	0.704
0.003	341	1.056
0.004	398	1.167

AUC_p53_ was integrated based on p53 levels that occurred between 0 and 500 time.

DNA damage occurred at time = 0.

In summary, we verified the relationships that exist between the DNA damage signal transduction, the cell cycle arrest and the induction of apoptosis by employing a seamless kinetic mathematical model that integrated several previously proven kinetic models. The numerical analysis also demonstrated that the dynamic behavior of p53 regulates the implementation of both the G2/M phase cell cycle arrest and the induction of apoptosis as follows: 1) Although the progression of G2/M phase cell cycle showed the retardation regardless of the initial DNA damage level, apoptosis was only induced when there was high DNA damage and the p53 time course exhibited an oscillation behavior; 2) Apoptosis induction needs a tremendous p53 activation with oscillation dynamics when there is high DNA damage; and 3) Apoptosis was implemented after the release of the G2/M phase cell cycle arrest. Therefore, when the p53 oscillation is observed, there is possibility that the cell implements the apoptosis induction. Moreover the apoptosis induction system is responsible for safeguarding the system that suppresses malignant transformations. As for the biological significance of the oscillation, an avoidance of overproduction of p53 should be worthy of special mention. Since p53 associates with several indispensable systems such as the cell cycle arrest, apoptosis induction and DNA repair, the overproduction of p53 has a grave effect on maintenance of homeostasis of cell. Although there was little difference between AUCp53 values when p53 showed sigmoidal behavior and oscillation (Table 1), the maximum p53 level increased with the initial DNA damage level (Table 2). The p53 ambidextrously controlled the implementation of both the cell cycle arrest system and apoptosis induction system with employing the diversity of own dynamic behaviors. Thus the oscillation contributed to avoid an overproduction of p53 and realized a sophisticated regulation of the biochemical systems. Such signal transduction is indispensable for not only practice of an effective utilization of resource but also realization of the complex systems in organism. The current study demonstrates that a numerical simulation that employs a seamless kinetic mathematical model can be used to elucidate and verify the dynamic behavior of a complex biological network. In the future, we hope to be able to identify several dominant factors associated with malignant transformations by applying a time-dependent sensitivity analysis [26] to our proposed model.

### Models

The p53 oscillation model [7], the G2/M phase cell cycle model [11] and the intrinsic apoptosis induction model [12] were constructed based on previous biological findings that made it possible to represent the dynamic behavior of biochemical species of interest. By applying these well-known kinetic mathematical models, we developed a novel model (our proposed model) that described the p53 oscillation system that is activated by DNA damage and which interferes with both the G2/M phase cell cycle progression and the induction of the intrinsic apoptosis. We then numerically analyzed the effects of p53's dynamic behavior for both the G2/M phase cell cycle arrest and the induction of the intrinsic apoptosis.

### p53 Oscillation System

Lev Bar-Or *et al.* was able to simulate the p53 oscillation dynamics by employing a simple kinetic mathematical model [7]. The model examined several biochemical species such as DNA damage, p53 and Mdm2 (Supporting information Figure S1). In this model, the II is considered to be a lumped dependent variable that creates a discrepancy for the peak time between p53 and Mdm2. The level of the DNA damage affects the synthesis processes of both p53 and II. p53 forms a negative feedback loop with Mdm2 via II, which results in an oscillation of the protein levels for both p53 and Mdm2. The functions of p53 are indispensable for the maintenance of both the genomic stability and the tissue homeostasis, which are preserved in a multitude of organisms in order to avoid malignant transformations. Therefore, p53 activates the synthesis processes of p21, 14-3-3 sigma and GADD45 on the G2/M phase of the cell cycle (Supporting information Figure S2), and causes interference between Bcl-2 and Bax that subsequently alters the induction of apoptosis (Supporting information Figure S3). By knowing the initial level of the DNA damage, it is possible to determine the protein level time courses based on this information. Therefore, this model can be used for a numerical analysis that evaluates the influence of the DNA damage on the dynamic behavior of p53, the cell cycle arrest and the induction of apoptosis.

### G2/M phase cell cycle arrest system

The principal biochemical species of the G2/M phase cell cycle are the M phase promoting factor (MPF), Cdc25 and Wee1. Since the central reaction involved is the activation of MPF by Cdc25 and Wee1 [27]–[28], any delay of the activation of MPF implies an arrest of the cell cycle. Aguda constructed a kinetic mathematical model (Aguad's model) that represented the dynamic behavior of biochemical species such as MPF, Cdc25 and Wee1 [11]. However, since Aguda's model did not take into consider the possibility of variations of the p53 oscillation dynamics, it is difficult to use the model to evaluate the relationship of the dynamic behavior of p53 and the cell cycle arrest. Consequently, we developed a kinetic mathematical model (Supporting information Figure S2) by integrating Aguda's model with the p53 oscillation system (Supporting information Figure S1). Our model was based on previous results that have shown that p21, 14-3-3 sigma and GADD45 were activated by p53, and thus, subsequently able to inhibit the activation of MPF [3]–[4]. p21 inhibits the cyclin:CDK complex, which has a central role in the cell cycle progression, and 14-3-3 sigma competitively binds to Cdc25, which is an activator of the MPF (cyclin B : Cdc2 complex : MPF). Moreover, GADD45 binds to Cdc2 and inhibits MPF synthesis [29]. Thus, DNA damage-caused activation of p53 temporarily results in cell cycle arrest, which simultaneously implements the DNA repair system [21], [30]. The kinetic mathematical model shown in supporting information Figure S2 was used to verify the relationship of the dynamic behavior of p53 in the arrest of the cell cycle.

### Intrinsic apoptosis induction system

The principal biochemical species of the intrinsic apoptosis induction are Bcl-2, Bax, Cyt_C_ and a series of caspases. Moreover, the central reaction that occurs involves the activation of the caspase cascade [23]. Bagci *et al*. constructed a kinetic mathematical model for the intrinsic apoptosis induction system. This model numerically presented the dynamic behavior of the caspase cascade that ultimately induces apoptosis [12]. However, since Bagci's model did not take the p53 oscillation dynamics into consideration, it is difficult to be able to evaluate the relationship of the dynamic behavior of p53 with the apoptosis induction. Consequently, we constructed a novel kinetic mathematical model (Supporting information Figure S3) that integrated Bagci's model with the p53 oscillation system (shown in supporting information Figure S1). In this model, p53 inhibits the Bcl-2 synthesis process, which normally suppresses the induction of apoptosis. In addition, there is activation of the Bax synthesis process, which is the trigger for the induction of the apoptosis. Bcl-2 is the mitochondrially-located apoptosis suppressor. It also has been shown to have a positive influence on the Bax degradation process [5]. Bax also promotes the excretion process that causes the transfer of Cytochrome C (Cyt_C_) from the mitochondria to the cellular cytoplasm [24]. Therefore, the activation of p53 causes a functional disorder of the reduction/oxidation reaction within the mitochondria, leading to the initiation of the caspase cascade, which is the downstream process of the apoptosis induction system. The increase in the caspase3 level that subsequently occurs implies that there is a completion of the apoptosis induction. Therefore, our proposed kinetic mathematical model can be used to verify the relationship of the dynamic behavior of p53 with the induction of apoptosis (Supporting information Figure S3).

### Numerical analysis

Using the above-mentioned kinetic mathematical models, we developed a novel kinetic model (proposed model) for which the p53 oscillation system that is activated by DNA damage simultaneously interferes in both the G2/M phase of the cell cycle and in the intrinsic apoptosis induction. Based on our proposed model combined with our use of the WinBEST-KIT, we were able to numerically obtain the protein level time courses [15]. The initial levels of the DNA damage were set between 0.0 and 0.004. These settings were used to create three categories, which consisted of no DNA damage (0.0), low DNA damage (0.001–0.002) and high DNA damage (0.003–0.004). In addition, the activity of p53 was evaluated by using the area under the curve (AUC), which is a well-established method that is used to evaluate the pharmacokinetics of the dose response. We numerically observed the time courses of the protein levels of interest, and verified the relationships between the p53 AUC, the peak times for the MPF levels, and the activation of the caspase cascade.

## Supporting Information

Table S1 (0.01 MB PDF)

Table S2 (0.03 MB PDF)

Table S3 (0.01 MB PDF)

Table S4 (0.01 MB PDF)

Figure S1 (0.02 MB PDF)

Figure S2 (0.03 MB PDF)

Figure S3 (0.06 MB PDF)

Figure S4 (0.03 MB PDF)

Figure S5 (0.04 MB PDF)

Figure S6 (0.04 MB PDF)

Figure S7 (0.03 MB PDF)

Figure S8 (0.04 MB PDF)

Figure S9 (0.05 MB PDF)

Figure S10 (0.04 MB PDF)
