# Supplementary material for: Sophisticated Framework between Cell Cycle Arrest and Apoptosis Induction Based on p53 Dynamics
Source: PLoS One. 2009 Mar 10;4(3):e4795. doi: 10.1371/journal.pone.0004795 (PMC2650779; doi:10.1371/journal.pone.0004795)
Supplement: Table S1 — (0.01 MB PDF) [file pone.0004795.s001.pdf]

Table S1 Dependent variables in the reaction scheme

p53 oscillation system

p53	p53	*DNA damage*	DNA damage
Mdm2	Mdm2	*Deg*	DNA damage degradation
II	intermediator II		

G2/M phase cell cycle arrest

Chk1	Chk1	*transducer*	DNA damage transducer
Chk1p	Chk1p	*p21*:*aMPF*	p21:aMPF complex
p21	p21	*iCdc25p*:*14_3_3*	iCdc25p:14-3-3 complex
Wee1	Wee1	*Wee1p*	Wee1p
iMPF	inactive MPF	*aMPF*	active MPF
aCdc25	active Cdc25	*aCdc25p*	active Cdc25p
iCdc25	inactive Cdc25	*iCdc25p*	inactive Cdc25p
14_3_3	14-3-3		

Apoptosis induction system

Apaf_1	Apaf-1	*Cytc*:*Apaf_1*	Cytochrome C:Apaf-1 complex
casp9	caspase9	$apop{:}pro9_2$,	apoptosome:procaspase9_2 complex
IAP	IAP	*casp9*:*IAP*	caspase9:IAP complex
pro9	procaspase9	*apop*:*pro9*	apoptosome:procaspase9 complex
apop	apoptosome	$apop{:}casp9_2$	apoptosome:caspase9_2
pro3	procaspase3	*apop*:*casp9*	apoptosome:caspase9 complex
casp8	caspase8	*apop*:*casp9*:*IAP*	apoptosome:caspase9:IAP complex
casp3	caspase3	*p21*:*Pro3*	p21:procaspase3 complex
Bid	Bid	$apop{:}casp9_2{:}IAP$	apoptosome:caspase9_2:IAP complex
Bcl_2	Bcl-2	*casp3*:*IAP*	caspase3:IAP complex
Bax	Bax	*casp9*:*pro3*	caspase9:pro3 complex
tBid	tBid	$apop{:}casp9_2{:}pro3$	apoptosome:caspase9_2:procaspase3
complex			
$Cytc_{mito}$	$\mathrm{Cytc}_{\mathrm{mito}}$	*casp8*:*Bid*	caspase8:Bid complex
Bax_2	Bax_2	*casp3*:*Bid*	caspase3:Bid complex
$tBid_{mito}$	$\mathrm{tBid}_{\mathrm{mito}}$	*casp3*:*Bcl_2*	caspase3:Bcl-2 complex
Cytc	Cytc	*tBid*:*Bax*	tBid:Bax complex
