# Supplementary material for: Sophisticated Framework between Cell Cycle Arrest and Apoptosis Induction Based on p53 Dynamics
Source: PLoS One. 2009 Mar 10;4(3):e4795. doi: 10.1371/journal.pone.0004795 (PMC2650779; doi:10.1371/journal.pone.0004795)
Supplement: Table S2 — (0.03 MB PDF) [file pone.0004795.s002.pdf]

Table S2 Mass balance equations

p53 oscillation system

$$\frac{dp53}{dt} = k_{1p}^{'} + k_{2p}^{'} \cdot \left(DNAdamage(0) \cdot \exp\left(-k_{4p}^{'} \cdot t\right)\right) - k_{3p}^{'} \cdot p53 - Deg(t) \cdot p53 \cdot Mdm2$$

$$\frac{dMdm2}{dt} = k_{10p}^{'} - k_{9p}^{'} \cdot Mdm2 + k_{8p}^{'} \cdot \frac{II^{9.0}}{Km^{9.0} + II^{9.0}}$$

$$\frac{dII}{dt} = k_{6p}^{'} \cdot p53 \cdot \frac{DNAdamage(0) \cdot \exp\left(-k_{4p}^{'} \cdot t\right)}{1.0 + k_{5p}^{'} \cdot p53 \cdot Mdm2} - k_{7p}^{'} \cdot II$$

$$Deg(t) = Deg(0) - k_{\deg} \cdot \left(DNAdamage(0) \cdot \exp\left(-k_{4p}^{'} \cdot t\right) - DNAdamage(0) \cdot \exp\left(-k_{damp} \cdot DNAdamage(0) \cdot t\right)\right)$$

G2/M phase cell cycle arrest

$$\frac{dChk1}{dt} = k_{4c}^{'} \cdot Chk1p - k_{3c}^{'} \cdot Chk1 \cdot transducer$$

$$\frac{dChk1p}{dt} = k_{3c}^{'} \cdot Chk1 \cdot transducer - k_{4c}^{'} \cdot Chk1p$$

$$\frac{dtransducer}{dt} = k_{1c}^{'} \cdot \left(DNAdamage(0) \cdot \exp\left(-k_{4p}^{'} \cdot t\right)\right) - k_{2c}^{'} \cdot transducer$$

$$\frac{diMPF}{dt} = \frac{k_{19c}^{'}}{1.0 + p53} + k_{21c}^{'} \cdot aMPF \cdot Wee1 - k_{20c}^{'} \cdot \left(aCdc25 + aCdc25p\right) \cdot iMPF$$

$$\frac{daMPF}{dt} = k_{20c}^{'} \cdot \left(aCdc25 + aCdc25p\right) \cdot iMPF + k_{23c}^{'} \cdot p21:aMPF - k_{21c}^{'} \cdot aMPF \cdot Wee1$$
$$- k_{24c}^{'} \cdot aMPF \cdot p21 - k_{22c}^{'} \cdot aMPF \cdot aMPF$$

$$\frac{dp21}{dt} = -k_{65a}^{'} \cdot p21 \cdot procaspase3 - k_{64a}^{'} \cdot p21 \cdot caspase3 + k_{66a}^{'} \cdot p21:procaspase3 + k_{25c}^{'} \cdot p53 + k_{26c}^{'}$$
$$+ k_{23c}^{'} \cdot p21:aMPF - k_{27c}^{'} \cdot p21 - k_{24c}^{'} \cdot aMPF \cdot p21$$

$$\frac{dp21:aMPF}{dt} = k_{24c}^{'} \cdot aMPF \cdot p21 - k_{23c}^{'} \cdot p21:aMPF$$

$$\frac{diCdc25}{dt} = k_{9c}^{'} \cdot aCdc25 + k_{8c}^{'} - k_{10c}^{'} \cdot aMPF \cdot iCdc25 - k_{11c}^{'} \cdot Chk1p \cdot iCdc25$$

$$\frac{diCdc25p}{dt} = k_{13c}^{'} \cdot aCdc25p + k_{11c}^{'} \cdot Chk1p \cdot iCdc25 - k_{12c}^{'} \cdot aMPF \cdot iCdc25p - k_{17c}^{'} \cdot iCdc25p \cdot 14\_3\_3$$

$$\frac{daCdc25p:14\_3\_3}{dt} = k_{17c}^{'} \cdot iCdc25p \cdot 14\_3\_3 - k_{18c}^{'} \cdot iCdc25:14\_3\_3$$

$$\frac{diCdc25}{dt} = k_{10c}^{'} \cdot aMPF \cdot iCdc25 + k_{7c}^{'} \cdot aCdc25p - k_{5c}^{'} \cdot aCdc25 - k_{6c}^{'} \cdot Chk1p \cdot aCdc25 - k_{9c}^{'} \cdot aCdc25$$

$$\frac{daCdc25p}{dt} = k_{6c}^{'} \cdot Chk1p \cdot aCdc25 + k_{12c}^{'} \cdot aMPF \cdot iCdc25p - k_{7c}^{'} \cdot aCdc25p - k_{13c}^{'} \cdot aCdc25p$$

$$\frac{d14\_3\_3}{dt} = k_{14c}^{'} \cdot p53 + k_{15c}^{'} - k_{17c}^{'} \cdot iCdc25p \cdot 14\_3\_3 - k_{16c}^{'} \cdot 14\_3\_3$$

$$\frac{dWee1}{dt} = k_{31c}^{'} + k_{29c}^{'} \cdot Wee1p - k_{28c}^{'} \cdot aMPF \cdot Wee1$$

$$\frac{dWee1p}{dt} = k_{28c}^{'} \cdot aMPF \cdot Wee1 - \left(k_{29c}^{'} + k_{30c}^{'}\right) \cdot Wee1p$$

Apoptosis induction system

$$\frac{dApaf\_1}{dt} = -k'_{26a} \cdot Cyt_C \cdot Apaf\_1 + k'_{27a} \cdot Cyt_C : Apaf\_1 + k'_{24a} - k'_{25a} \cdot Apaf\_1$$

$$\frac{dCyt_C : Apaf\_1}{dt} = k'_{26a} \cdot Cyt_C \cdot Apaf\_1 - k'_{27a} \cdot Cyt_C : Apaf\_1 - 7.0 \cdot \left(k'_{28a} \cdot Cyt_C : Apaf\_1^p + k'_{29a} \cdot apoptosome\right)$$

$$\frac{dapoptosome}{dt} = k'_{28a} \cdot Cyt_C : Apaf\_1^p - k'_{29a} \cdot apoptosome - k'_{34a} \cdot apoptosome \cdot procaspase9$$
$$+ k'_{33a} \cdot apoptosome : procaspase9 + k'_{32a} \cdot apoptosome : caspase9 - k'_{31a} \cdot apoptosome \cdot caspase9$$

$$\frac{dapoptosome : procaspase9}{dt} = k'_{34a} \cdot apoptosome \cdot procaspase9 - k'_{33a} \cdot apoptosome : procaspase9$$
$$- k'_{35a} \cdot apoptosome : procaspase9 \cdot procaspase9 + k'_{36a} \cdot apoptosome : procaspase9_2$$

$$\frac{dapoptosome : procaspase9_2}{dt} = k'_{35a} \cdot apoptosome : procaspase9 \cdot procaspase9$$
$$- k'_{36a} \cdot apoptosome : procaspase9_2 - k'_{39a} \cdot apoptosome : procaspase9_2$$

$$\frac{dapoptosome : caspase9_2}{dt} = k'_{39a} \cdot apoptosome : procaspase9_2 - k'_{38a} \cdot apoptosome : caspase9_2$$
$$+ k'_{37a} \cdot apoptosome : caspase9 \cdot caspase9 - k'_{49a} \cdot apoptosome : caspase9_2 \cdot IAP$$
$$+ k'_{50a} \cdot apoptosome : caspase9_2 : IAP - k'_{57a} \cdot apoptosome : caspase9_2 \cdot procaspase3$$
$$+ k'_{58a} \cdot apoptosome : caspase9_2 : procaspase3 + k'_{41a} \cdot apoptosome : caspase9_2 : procaspase3$$

$$\frac{dapoptosome : caspase9}{dt} = k'_{38a} \cdot apoptosome : caspase9_2 - k'_{37a} \cdot apoptosome : caspase9 \cdot caspase9$$
$$- k'_{32a} \cdot apoptosome : caspase9 + k'_{31a} \cdot apoptosome \cdot caspase9$$
$$- k'_{48a} \cdot apoptosome : caspase9 \cdot IAP + k'_{47a} \cdot apoptosome : caspase9 : IAP$$

$$\frac{dcaspase9}{dt} = k'_{38a} \cdot apoptosome : caspase9_2 - k'_{37a} \cdot apoptosome : caspase9 \cdot caspase9$$
$$+ k'_{32a} \cdot apoptosome : caspase9 - k'_{31a} \cdot apoptosome \cdot caspase9 - k'_{51a} \cdot caspase9 \cdot IAP$$
$$+ k'_{52a} \cdot caspase9 : IAP - k'_{45a} \cdot caspase9 \cdot procaspase3 + k'_{46a} \cdot caspase9 : procaspase3$$
$$+ k'_{44a} \cdot caspase9 : procaspase3 - k'_{69a} \cdot caspase9$$

$$\frac{dprocaspase9}{dt} = -k'_{34a} \cdot apoptosome \cdot procaspase9 + k'_{33a} \cdot apoptosome : procaspase9$$
$$- k'_{35a} \cdot apoptosome : procaspase9 \cdot procaspase9 + k'_{36a} \cdot apoptosome : procaspase9_2$$
$$+ k'_{40a} - k'_{30a} \cdot procaspase9$$

$$\frac{dIAP}{dt} = -k'_{51a} \cdot caspase9 \cdot IAP + k'_{52a} \cdot caspase9 : IAP - k'_{48a} \cdot apoptosome : caspase9 \cdot IAP$$
$$+ k'_{47a} \cdot apoptosome : caspase9 : IAP - k'_{49a} \cdot apoptosome : caspase9_2 \cdot IAP$$
$$+ k'_{50a} \cdot apoptosome : caspase9_2 : IAP - k'_{56a} \cdot caspase3 \cdot IAP$$
$$+ k'_{55a} \cdot caspase3 : IAP + k'_{53a} - k'_{54a} \cdot IAP$$

$$\frac{dcaspase9 : IAP}{dt} = k'_{51a} \cdot caspase9 \cdot IAP - k'_{52a} \cdot caspase9 : IAP$$

$$\frac{dapoptosome : caspase9 : IAP}{dt} = k'_{48a} \cdot apoptosome : caspase9 \cdot IAP - k'_{47a} \cdot apoptosome : caspase9 : IAP$$

$$\frac{dapoptosome : caspase9_2 : IAP}{dt} = k'_{49a} \cdot apoptosome : caspase9_2 \cdot IAP - k'_{50a} \cdot apoptosome : caspase9_2 : IAP$$

Apoptosis induction system (continued)

$$\frac{dcaspase3:IAP}{dt} = k'_{56a}\cdot caspase3\cdot IAP - k'_{55a}\cdot caspase3:IAP$$

$$\frac{dprocaspase3}{dt} = -k'_{65a}\cdot p21\cdot procaspase3 + k'_{66a}\cdot p21:procaspase3 - k'_{45a}\cdot caspase9\cdot procaspase3$$
$$+ k'_{46a}\cdot caspase9:procaspase3 - k'_{57a}\cdot apoptosome:caspase9_2:procaspase3$$
$$+ k'_{58a}\cdot apoptosome:caspase9_2:procaspase3 + k'_{42a} - k'_{43a}\cdot procaspase3$$

$$\frac{dcaspase9:procaspase3}{dt} = k'_{45a}\cdot caspase9\cdot procaspase3 - k'_{46a}\cdot caspase9:procaspase3$$
$$- k'_{44a}\cdot caspase9:procaspase3$$

$$\frac{dapoptosome:caspase9_2:procaspase3}{dt} = k'_{57a}\cdot apoptosome:caspase9_2\cdot procaspase3$$
$$- k'_{58a}\cdot apoptosome:caspase9_2:procaspase3 - k'_{41a}\cdot apoptosome:caspase9_2:procaspase3$$

$$\frac{dcaspase3}{dt} = k'_{44a}\cdot caspase9:procaspase3 + k'_{41a}\cdot apoptosome:caspase9_2:procaspase3$$
$$- k'_{56a}\cdot caspase3\cdot IAP + k'_{55a}\cdot caspase3\cdot IAP - k'_{68a}\cdot caspase3\cdot Bid + \left(k'_{59a} + k'_{67a}\right)\cdot caspase3:Bid$$
$$- k'_{61a}\cdot caspase3\cdot Bcl\_2 + \left(k'_{60a} + k'_{62a}\right)\cdot caspase3:Bcl\_2 - k'_{63a}\cdot caspase3$$

$$\frac{dcaspase8}{dt} = -k'_{3a}\cdot caspase8\cdot Bid + k'_{2a}\cdot caspase8:Bid + k'_{4a}\cdot caspase8:Bid - k'_{1a}\cdot caspase8$$

$$\frac{dBid}{dt} = -k'_{3a}\cdot caspase8\cdot Bid + k'_{2a}\cdot caspase8:Bid - k'_{68a}\cdot caspase3\cdot Bid$$
$$+ k'_{67a}\cdot caspase3:Bid + k'_{6a} - k'_{5a}\cdot Bid$$

$$\frac{dcaspase8:Bid}{dt} = k'_{3a}\cdot caspase8\cdot Bid - k'_{2a}\cdot caspase8:Bid - k'_{4a}\cdot caspase8:Bid$$

$$\frac{dBcl\_2}{dt} = -k'_{61a}\cdot caspase3\cdot Bcl\_2 + k'_{60a}\cdot caspase3:Bcl\_2 - k'_{13a}\cdot Bcl\_2\cdot Bax$$
$$+ \frac{k'_{10a}\cdot p53_{threshold}^{4.0}}{p53^{4.0} + p53_{threshold}^{4.0}} - k'_{11a}\cdot Bcl\_2$$

$$\frac{dcaspase3:Bid}{dt} = k'_{68a}\cdot caspase3\cdot Bid - k'_{67a}\cdot caspase3:Bid - k'_{59a}\cdot caspase3:Bid$$

$$\frac{dcaspase3:Bcl\_2}{dt} = k'_{61a}\cdot caspase3\cdot Bcl\_2 - k'_{60a}\cdot caspase3:Bcl\_2 - k'_{62a}\cdot caspase3:Bcl\_2$$

$$\frac{dBax}{dt} = -k'_{15a}\cdot tBid_{mito}\cdot Bax - k'_{18a}\cdot tBid:Bax\cdot Bax - k'_{13a}\cdot Bcl\_2\cdot Bax$$
$$+ \frac{k'_{12a}\cdot\left(1.0 + p53^{4.0}\right)}{p53^{4.0} + p53_{threshold}^{4.0}} - k'_{14a}\cdot Bax$$

$$\frac{dtBid}{dt} = k'_{4a}\cdot caspase8:Bid + k'_{59a}\cdot caspase3:Bid - k'_{8a}\cdot tBid + k'_{18a}\cdot tBid:Bax\cdot Bax - k'_{7a}\cdot tBid$$

$$\frac{dtBid:Bax}{dt} = k'_{15a}\cdot tBid_{mito}\cdot Bax - k'_{18a}\cdot tBid:Bax\cdot Bax - k'_{17a}\cdot tBid:Bax$$

$$\frac{dCyt_{Cmito}}{dt} = -k'_{22a}\cdot Bax_2\cdot Cyt_{Cmito} + k'_{20a} - k'_{21a}\cdot Cyt_{Cmito}$$

$$\frac{dBax_2}{dt} = k'_{18a}\cdot tBid:Bax\cdot Bax - k'_{19a}\cdot Bax_2$$

Apoptosis induction system (continued)

$$\frac{dtBid_{mito}}{dt} = k'_{8a} \cdot tBid - k'_{15a} \cdot tBid_{mito} \cdot Bax - k'_{9a} \cdot tBid_{mito}$$

$$\frac{dCyt_C}{dt} = k'_{22a} \cdot Bax_2 \cdot Cyt_{Cmito} - k'_{26a} \cdot Cyt_C \cdot Apaf\_1 + k'_{27a} \cdot Cyt_C : Apaf\_1 - k'_{23a} \cdot Cyt_C$$

$$\frac{dp21 : procaspase3}{dt} = k'_{65a} \cdot p21 \cdot procaspase3 - k'_{66a} \cdot p21 : procaspase3$$
