# Supplementary material for: Sophisticated Framework between Cell Cycle Arrest and Apoptosis Induction Based on p53 Dynamics
Source: PLoS One. 2009 Mar 10;4(3):e4795. doi: 10.1371/journal.pone.0004795 (PMC2650779; doi:10.1371/journal.pone.0004795)
Supplement: Table S3 — (0.01 MB PDF) [file pone.0004795.s003.pdf]

Table S3 Initial conditions

p53 oscillation system
p53(0)=0.0001, *Mdm2*(0)=0.5875e-4, *II*(0)=0.0, *Deg*(0)=0.0556
G2/M phase cell cycle arrest
Chk1p(0)=2.5e-7, *Chk1*(0)=0.24999975, *transducer*(0)=0.05, *iMPF*(0)=0.25e-6, *aMPF*(0)=0.25e-8, *p21*(0)=0.0, *p21*:*aMPF*(0)=0.0, *Wee1p*(0)=0.0, *iCdc25*(0)=0.25e-6, *iCdc25Ps216*(0)=0.5e-5, *iCdc25P*:*14_3_3*(0)=0.0075, *aCdc25*(0)=0.25e-6, *aCdc25Ps216*(0)=0.0, *14-3-3*(0)=0.5, *Wee1*(0)=0.00025
Apoptosis induction system
Apaf_1(0)=0.004, Cyt_C:*Apaf_1*(0)=0.0, *apoptosome*(0)=0.0, *apoptosome*:*procaspase9*(0)=0.0, *apoptosome*:$procaspase9_2$(0)=0.0, *apoptosome*:$caspase9_2$(0)=0.0, *apoptosome*:*caspase9*(0)=0.0, *caspase9*(0)=0.0, *procaspase9*(0)=0.004, *IAP*(0)=0.004, *caspase9*:*IAP*(0)=0.0, *apoptosome*:*caspase9*:*IAP*(0)=0.0, *apoptosome*:$caspase9_2$:*IAP*(0)=0.0, *caspase3*:*IAP*(0)=0.0, *procaspase3*(0)=0.004, *caspase9*:*procaspase3*(0)=0.0, *apoptosome*:$caspase9_2$:*procaspase3*(0)=0.0, *caspase3*(0)=0.00001, *caspase8*(0)=0.0001, *Bid*(0)=0.004, *caspase8*:*Bid*(0)=0.0, *Bcl_2*(0)=0.004, *caspase3*:*Bid*(0)=0.0, *caspase3*:*Bcl_2*(0)=0.0, *Bax*(0)=0.004, *tBid*(0)=0.0, *tBid*:*Bax*(0)=0.0, Cyt_{Cmito}(0)=0.004, Bax_2(0)=0.0, $tBid_{mito}$(0)=0.0, Cyt_C(0)=0.0, *p21*:*procaspase3*(0)=0.0
