# Supplementary material for: Sophisticated Framework between Cell Cycle Arrest and Apoptosis Induction Based on p53 Dynamics
Source: PLoS One. 2009 Mar 10;4(3):e4795. doi: 10.1371/journal.pone.0004795 (PMC2650779; doi:10.1371/journal.pone.0004795)
Supplement: Table S4 — (0.01 MB PDF) [file pone.0004795.s004.pdf]

Table S4 Kinetic parameters

p53 Oscillation system
k'_{1p}=0.000025, k'_{2p}=1.5, k'_{3p}=0.001, k'_{4p}=1.0E-8, k'_{5p}=0.004, k'_{6p}=6.0, k'_{7p}=0.005, k'_{8p}=10.0, k'_{9p}=0.02, k'_{10p}=0.00094, k_{ex}=1.0, Km=9.5, k_{damp}=0.02, k_{deg}=0.772
G2/M phase cell cycle arrest
k'_{1c}=0.2, k'_{2c}=1.0, k'_{3c}=1.0, k'_{4c}=1.0, k'_{5c}=0.0001, k'_{6c}=0.001, k'_{7c}=0.01, k'_{8c}=1.0e-5, k'_{9c}=0.01, k'_{10c}=1.0, k'_{11c}=0.01, k'_{12c}=1.0, k'_{13c}=0.01, k'_{14c}=0.01, k'_{15c}=1.0, k'_{16c}=1.0, k'_{17c}=100.0, k'_{18c}=1.0, k'_{19c}=0.005, k'_{20c}=1.0, k'_{21c}=1.0, k'_{22c}=0.01, k'_{23c}=1.0, k'_{24c}=0.1, k'_{25c}=0.1, k'_{26c}=0.01, k'_{27c}=1.0, k'_{28c}=0.1, k'_{29c}=1.0, k'_{30c}=1.0, k'_{31c}=2.0E-4 4
Apoptosis induction system
k'_{1a}=0.006, k'_{2a}=0.5, k'_{3a}=10.0, k'_{4a}=0.1, k'_{5a}=0.006, k'_{6a}=0.00003, k'_{7a}=0.006, k'_{8a}=10.0, k'_{9a}=0.006, k'_{10a}=8.0E-5, k'_{11a}=0.006, k'_{12a}=3.0E-5, k'_{13a}=10.0, k'_{14a}=0.006, k'_{15a}=10.0, k'_{17a}=0.006, k'_{18a}=10.0, k'_{19a}=0.006, k'_{20a}=0.0003, k'_{21a}=0.006, k'_{22a}=10.0, k'_{23a}=0.006, k'_{24a}=0.0003, k'_{25a}=0.006, k'_{26a}=5.0, k'_{27a}=0.5, k'_{28a}=5.0E+4, k'_{29a}=0.5, k'_{30a}=0.006, k'_{31a}=0.5, k'_{32a}=5.0, k'_{33a}=0.5, k'_{34a}=10.0, k'_{35a}=10.0, k'_{36a}=0.5, k'_{37a}=0.5, k'_{38a}=5.0, k'_{39a}=0.1, k'_{40a}=0.0003, k'_{41a}=0.1, k'_{42a}=0.0003, k'_{43a}=0.006, k'_{44a}=0.001, k'_{45a}=10.0, k'_{46a}=0.5, k'_{47a}=0.0035, k'_{48a}=5.0, k'_{49a}=5.0, k'_{50a}=0.0035, k'_{51a}=5.0, k'_{52a}=0.0035, k'_{53a}=3.0E-5, k'_{54a}=0.006, k'_{55a}=0.0035, k'_{56a}=5.0, k'_{57a}=10.0, k'_{58a}=0.5, k'_{59a}=0.1, k'_{60a}=10.0, k'_{61a}=0.5, k'_{62a}=0.1, k'_{63a}=0.006, k'_{64a}=1.0, k'_{65a}=0.1, k'_{66a}=1.0, k'_{67a}=0.5, k'_{68a}=10.0, k'_{69a}=0.006, $p53_{threshold}$=0.4, p=4.0
