# Supplementary material for: Sophisticated Framework between Cell Cycle Arrest and Apoptosis Induction Based on p53 Dynamics
Source: PLoS One. 2009 Mar 10;4(3):e4795. doi: 10.1371/journal.pone.0004795 (PMC2650779; doi:10.1371/journal.pone.0004795)
Supplement: Figure S1 — (0.02 MB PDF) [file pone.0004795.s005.pdf]

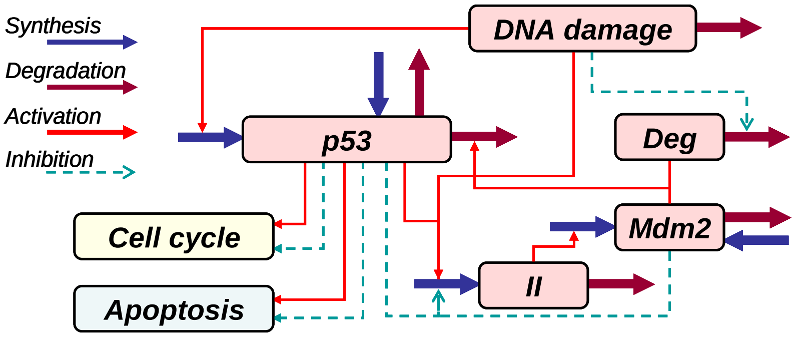

Figure S1. p53 oscillation system reaction scheme.

Blue and russet thick arrows represent the synthetic and degradation process, respectively. Red arrows and dashed green arrows are activation and suppression, respectively. The kinetic parameters for each of the processes are shown in Supporting information Figure S4.
