# Supplementary material for: Sophisticated Framework between Cell Cycle Arrest and Apoptosis Induction Based on p53 Dynamics
Source: PLoS One. 2009 Mar 10;4(3):e4795. doi: 10.1371/journal.pone.0004795 (PMC2650779; doi:10.1371/journal.pone.0004795)
Supplement: Figure S2 — (0.03 MB PDF) [file pone.0004795.s006.pdf]

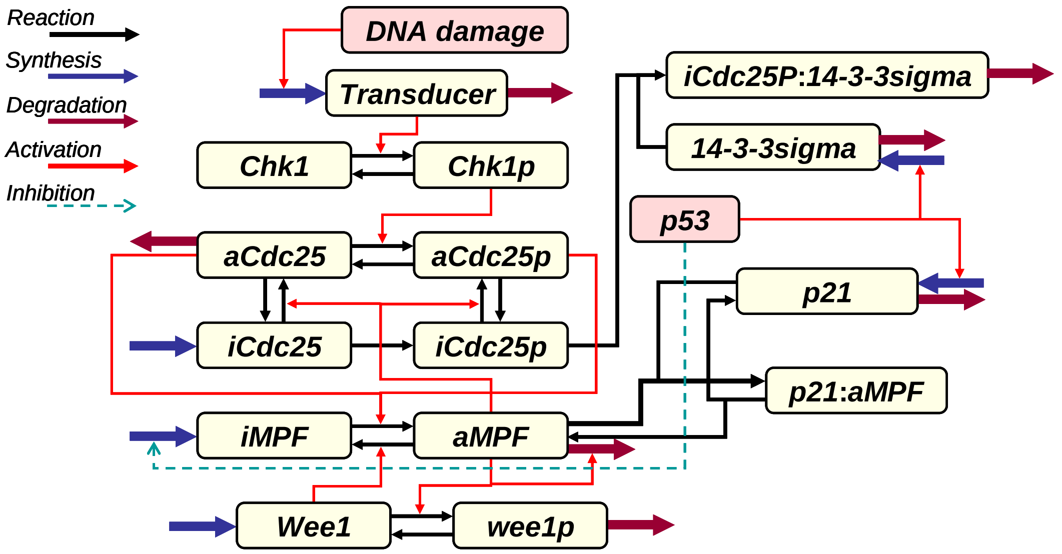

Figure S2. G2/M phase cell cycle arrest reaction scheme.

Blue and russet thick arrows represent the synthetic and degradation process, respectively. Black and red arrows show the reaction and activation, respectively, while the dashed arrow indicates suppression. The kinetic parameters for each of the processes are shown in Supporting information Figures S5 and S6.
