# Supplementary material for: Sophisticated Framework between Cell Cycle Arrest and Apoptosis Induction Based on p53 Dynamics
Source: PLoS One. 2009 Mar 10;4(3):e4795. doi: 10.1371/journal.pone.0004795 (PMC2650779; doi:10.1371/journal.pone.0004795)
Supplement: Figure S3 — (0.06 MB PDF) [file pone.0004795.s007.pdf]

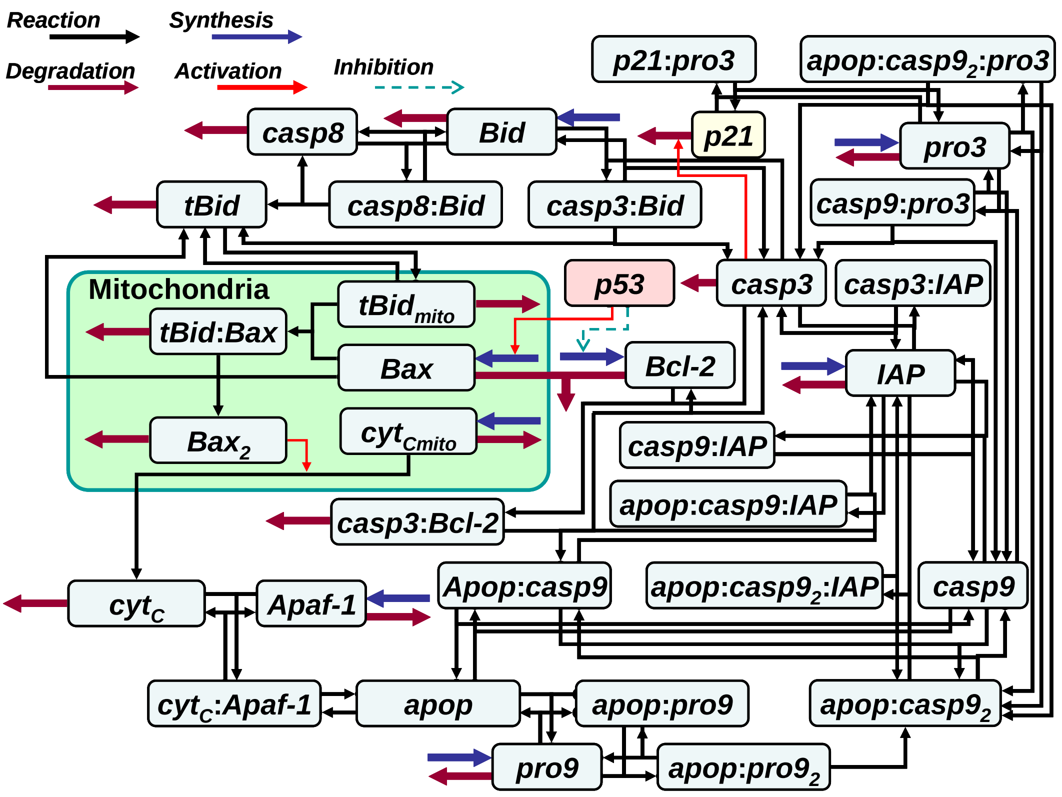

Figure S3. Apoptosis induction system reaction scheme.

Blue and russet thick arrows represent the synthetic and degradation process, respectively. Black and red arrows show the reaction and activation, respectively, while the dashed arrow indicates suppression. The kinetic parameters for each of the processes are shown in the Supporting information Figures S7 and S10.
