# Supplementary material for: Sophisticated Framework between Cell Cycle Arrest and Apoptosis Induction Based on p53 Dynamics
Source: PLoS One. 2009 Mar 10;4(3):e4795. doi: 10.1371/journal.pone.0004795 (PMC2650779; doi:10.1371/journal.pone.0004795)
Supplement: Figure S10 — (0.04 MB PDF) [file pone.0004795.s014.pdf]

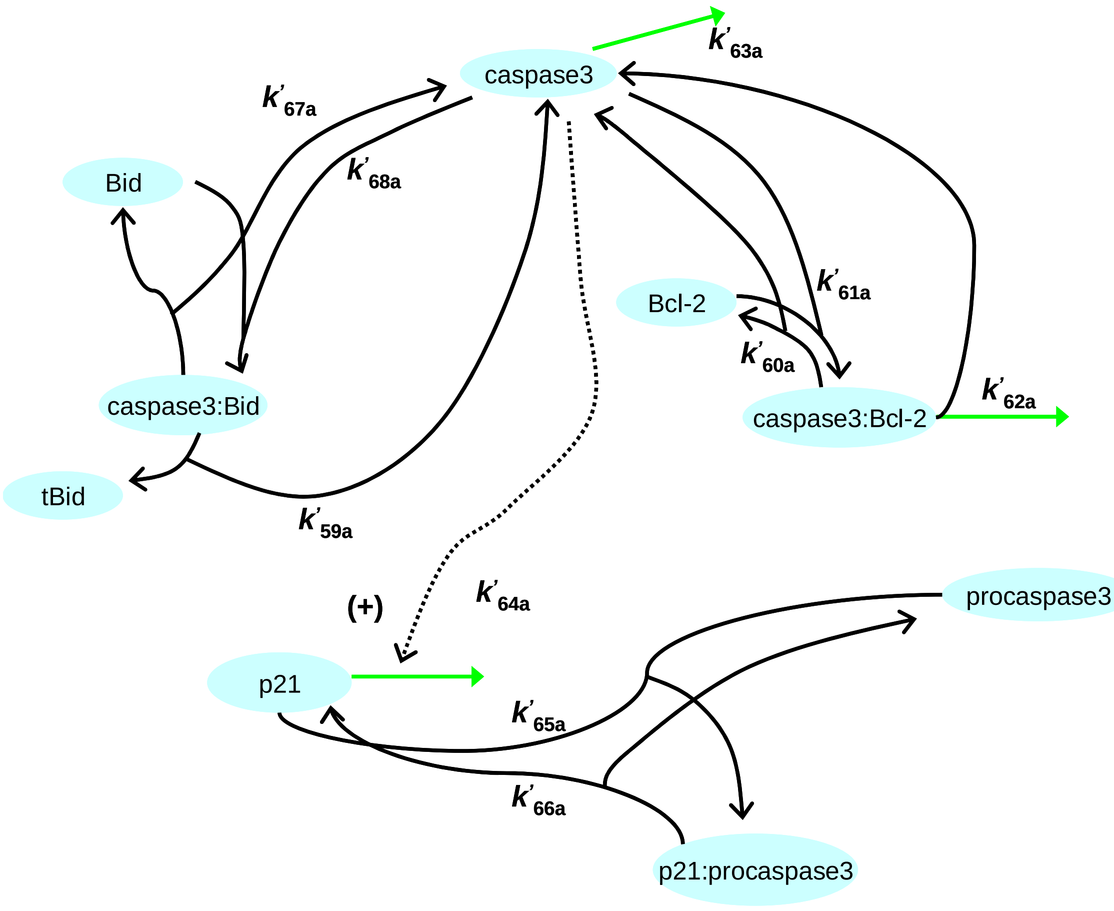

Figure S10. Regulation of caspase3 in the apoptosis induction system.

Green thick arrows represent the degradation process. Black arrows show the reaction process. Dashed arrows with a plus sign represent a positive interaction.
